# Synchronous Fibrolamellar Hepatocellular Carcinoma and Auricular Myxoma

**DOI:** 10.1155/2015/241708

**Published:** 2015-10-05

**Authors:** Yessica M. González-Cantú, Cristina Rodriguez-Padilla, Martha Lilia Tena-Suck, Alberto García de la Fuente, Rosa María Mejía-Bañuelos, Raymundo Díaz Mendoza, Samuel Quintanilla-Garza, Yolaester Batisda-Acuña

**Affiliations:** ^1^Laboratorio de Inmunología y Virología, Facultad de Ciencias Biológicas, Universidad Autónoma de Nuevo León, Avenida Manuel L. Barragán S/N, Ciudad Universitaria, 66451 San Nicolás de los Garza, NL, Mexico; ^2^Departamento de Neuropatología, Instituto Nacional de Neurología y Neurocirugía, Manuel Velasco Suárez, Avenida Insurgentes Sur 3877, Colonia La Fama, 14269 Delegación Tlalpan, DF, Mexico; ^3^Hospital General de Zona 17, Instituto Mexicano del Seguro Social, Fortunato Lozano y Roble Colonia Benito Juárez, 64420 Monterrey, NL, Mexico; ^4^Unidad de Medicina Familiar No. 73, Instituto Mexicano del Seguro Social, Pablo de Mejía No. 526, Colonia Zona Centro, 25000 Saltillo, COAH, Mexico; ^5^Hospital General Regional No. 1, Instituto Mexicano del Seguro Social, Bosques de los Olivos 101, 61301 Morelia, MICH, Mexico

## Abstract

Synchronic occurrence of benign and malignant tumors is extremely rare. Fibrolamellar hepatocellular carcinoma represents 1% to 2% of all hepatocarcinomas, while myxomas represent about half of all the cases of primary tumors of the heart. We present the case of a 53-year-old woman with a left atrial myxoma that was surgically removed. Several weeks later, the patient returned to the hospital with abdominal pain. CT scan showed a mass in the left lobe of the liver that was resected and diagnosed as fibrolamellar hepatocellular carcinoma. As of this writing, the patient is healthy.

## 1. Introduction

Hepatocellular carcinoma (HCC) represents 80% to 90% of all liver cancers [1]. According to the International Classification of Disease for Oncology, HCC is categorized as HCC-not otherwise specified (HCC-NOS), scirrhous HCC, spindle cell variant HCC, clear cell HCC, pleomorphic HCC, and fibrolamellar HCC (FLHCC) [2]. FLHCC is a relatively rare form of HCC that affects mostly young people and is not associated with any diseases of the liver such as cirrhosis or hepatitis virus infection [3]. FLHCC is less aggressive than HCC [4].

Primary cardiac tumors are rare, representing 0.0017% to 0.19% of autopsy cases [5]. Myxomas represent about 50% of all cardiac tumors [6] and are located mostly in the left atrium (left-right ratio = 4 : 1) [5]. Most cardiac myxomas are sporadic, although a small percentage of them are familial and sometimes attributable to Carney syndrome.

Here we describe the case of a 53-year-old woman who was diagnosed with fibrolamellar hepatocellular carcinoma synchronous with left atrial myxoma.

## 2. Case Presentation

A 53-year-old female with no known medical conditions reported the start of symptoms 4 months before consultation. The patient reported palpitations that exacerbated by cardiovascular exercise and attenuated with rest. The cardiologist requested an ECG and diagnosed sinus tachycardia. Subsequently, a transthoracic echocardiography was performed, and a mass dependent on the left atrium compatible with auricular myxoma was detected. The patient was scheduled for surgery, a median sternotomy for a transseptal approach to the right atrium was performed, and the patient began treatment with IMMUNEPOTENT CRP (bovine dialyzable leukocyte extract) which was administered orally to promote postsurgical recovery.

**Figure 1:**
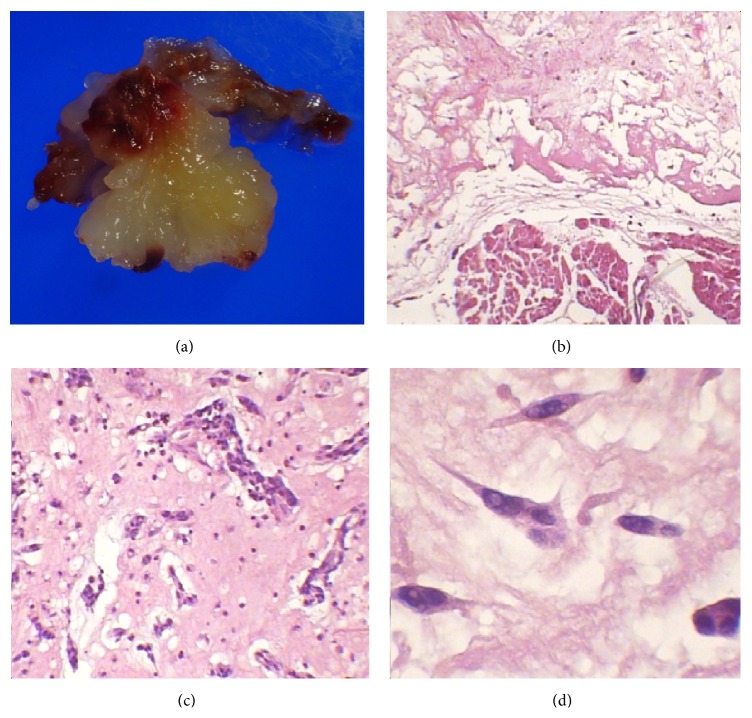
(a) Macroscopic image of the left auricular myxoma. (b) shows fusiform hyperchromatic cells immersed in a myxoid matrix and the cardiac muscle (H and E stain, 50x). (c) Groups of fusiform cells immersed in a myxoid matrix surrounded by abundant inflammatory cells (H and E stain, 100x). (d) Fusiform cells under higher magnification (H and E stain, 400x).

The patient underwent surgery without complications and was discharged from the hospital. The mass was diagnosed by pathology as left atrial myxoma with mural thrombus (Figure 1). Three months after discharge from the hospital, the patient presented epigastric abdominal pain of moderate intensity that irradiated to the mesogastrium and right upper quadrant, which was accompanied by postprandial pain, nausea, and constipation. The patient was admitted to the hospital to evaluate her abdominal pain. Her heart rate was 100 bpm and her blood pressure was 130/80 mmHg; all laboratory tests were normal.

**Figure 2:**
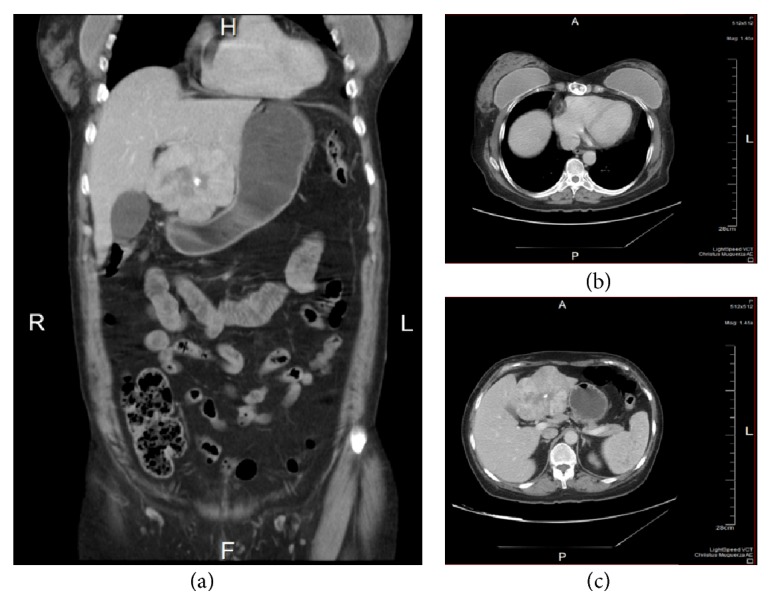
Images from the abdominal CT. (a) and (b) show the 8 × 8 × 7.3 cm tumor located in segment IV of the left lobe of the liver that laterally displaces the gallbladder. The lesion has defined lobular borders that show a heterogeneous peripheral areas and a hypodense center with calcification. (c) shows changes in the sternum with presence of surgical material.

A CT scan of the abdomen was performed, which showed 8 × 8 × 7.3 cm mass in the left lobe of the liver (Figure 2(a)). The lesion was hypervascular and had central calcification and a tendency toward necrosis (Figures 2(b) and 2(c)). The patient underwent surgery for tumor resection.

**Figure 3:**
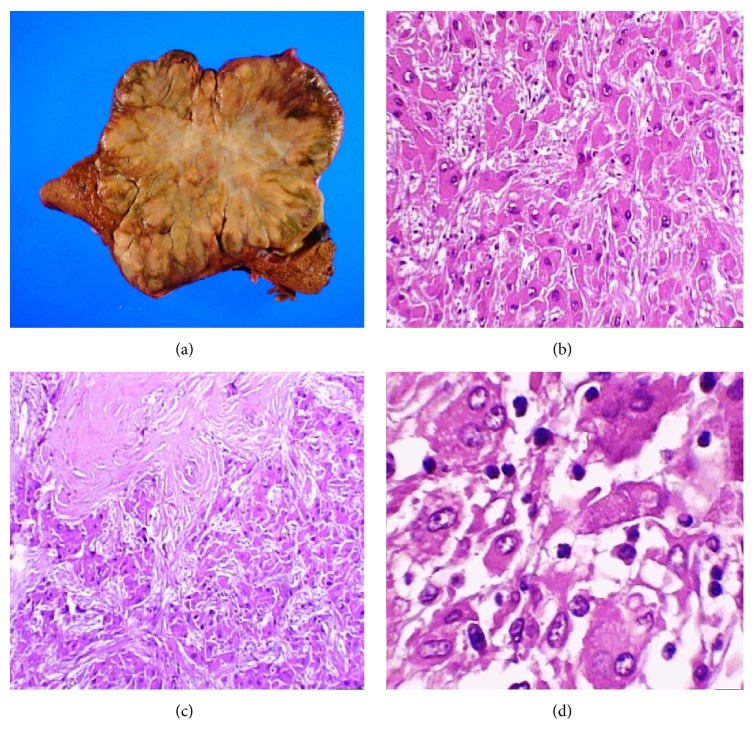
(a) Macroscopic image of the liver that shows a 9 × 7 cm, central yellowish lesion with diffuse, infiltrating borders with a whitish central lesion. (b) shows neoplastic cells separated into nodules by connective tissue (H and E stain, 50x). (c) Neoplastic cells with loss of the nucleus-cytoplasm relationship (H and E stain, 100x). (d) Image that shows the cellular and nuclear pleomorphism in greater detail (H and E stain, 400x).

After postoperative analysis from pathology, the diagnosis was of a 9 cm, Grade III moderately differentiated fibrolamellar hepatocellular carcinoma with clean resection borders (AJCC, T2, N0, Mx) (Figures 3(a)–3(d)). The patient is currently asymptomatic and receives annual check-ups.

## 3. Discussion

Multiple primary malignant neoplasms (MPMN) are defined as a diagnosis of two or more independent primary malignancies of different histologies/origins in an individual [7]. They are classified as MPMN according to the following criteria: tumors must be malignant, they must be located in different organs or tissues, and the possibility of metastasis must be ruled out completely. The incidence rate of MPNM varies from 0.7% to 11.7% [8]. The association of benign and malignant tumors, in absence of underlying genetic diseases, is extremely rare [9].

Fibrolamellar hepatocellular carcinoma and cardiac myxoma are a rare presentation of synchronous tumors. Ochoa et al. (2011) described one case of hepatocarcinoma synchronous with atrial myxoma [9]. There at least two cases of metastasis mimicking atrial myxoma in hepatocellular carcinoma [1, 10]. Fibrolamellar hepatocellular carcinoma (FLHCC) usually appears in younger patients (median age of 25 years) [11], although recent studies show two distinct peaks of incidence, the first at 10 to 30 years of age and the second at 70 to 79 years of age [2, 4]. At the moment of diagnosis, our patient was 53 years old, which is inconsistent with the data reported in the literature.

FLHCC is usually asymptomatic, and most patients visit a doctor when they note the presence of an abdominal mass. Those patients with symptomatology report vague symptoms such as weight loss and abdominal pain or discomfort [3]. Imaging studies, such as ultrasound, MRI, and CT scans, are an integral part of FLHCC diagnosis [4]. Our patient was diagnosed with FLHCC during a CT scan performed a few weeks after surgery to remove myxoma.

Liver function tests for patients with FLHCC are usually normal, although sometimes alpha-fetoprotein and aminotransferases show slightly increased levels [3, 12].

FHLCC prefers the left lobe of the liver, and, grossly, it is usually yellow to pale tan and firm, with an average size of 6–19 cm at its longest diameter. About 75% of tumors present a central scar; some tumors present foci of necrosis, hemorrhage, and calcification [3, 11].

Histopathologically, the tumor is composed of large polygonal cells with large vesicular nuclei containing marginalized chromatin and prominent nucleoli with eosinophilic (oncocytic) cytoplasm surrounded by distinctly lamellar stroma. Calcifications and pale bodies are seen in more than 50% of cases [11, 13]. Stainable copper is found in 75% of all FLHCC cases [12]. Immunohistochemical markers for FHLCC are CK7, EMA, mCEA, CA19-9, EpCAM, and CD68 [11, 13]. Whenever possible, complete surgical resection is the preferred treatment for FLHCC [4].

Myxomas represent 50% of the very rare cases of primary cardiac tumors. The most frequent location for myxomas is the left atrium, and they usually originate from the endocardium of the atrial septum [14, 15]. Generally, myxomas appear in women aged 30 to 70 years. Clinically, myxomas are characterized by dyspnea, syncope, arrhythmias, edema, hemoptysis, and sudden death. Some of the systemic characteristics of myxoma include embolic phenomena in 30% to 40% of patients. Approximately 10% to 15% of patients are asymptomatic and the myxoma is found accidentally during imaging studies [16]. Transthoracic or transesophageal echocardiography and MRI are the most common diagnostic tools for neoplasms of the heart [14]. In our case, the patient reported tachycardia, which led to the discovery of the myxoma by means of transesophageal echocardiography.

Surgery is the election treatment for myxoma; it must be performed without delay because of the high risk of sudden death from thromboembolism or valvular obstruction; resection of the tumor must be total [14, 17].

Macroscopically, myxomas are pedunculated and gelatinous, and their surface can be smooth, villous, or friable and, in some cases, they show areas of hemorrhage. Their diameter ranges from 1 to 15 cm, and their weight ranges between 15 and 180 grams [5, 18].

Histologically, myxomas are made up of stellate and polyhedral (lepidic) cells. These tumor cells have small round or oval nuclei and variable amounts of cytoplasm. Myxoma cells can appear singly or in small clusters or chords, and when grouped around small spaces they can simulate glandular structures; they rarely show mitotic activity. Myxomas have abundant, fibrillary, slightly basophilic, mucoid matrix [19]. These tumors may form large and small fibrin-platelet thrombi, which may be an additional source of emboli [19].

Myxomas show a diffuse positive reaction for the immunohistochemical marker Vimentin and focal expression of CD34, CD68, and SMA [20]. They also have variable positivity for cytokeratin, S100, factor VIII, and Calretinin [5, 20].

The IMMUNEPOTENT CRP (bovine dialyzable leukocyte extract) is a mixture of low molecular weight substances and is a drug capable of modulating the immune response. It has been used for years in clinical trials of multiple types of diseases such as cancer, toxic shock, and postoperative recovery, as in the clinical case presented here, increasing the quality of life of the patients [21, 22].

Synchronous tumors are rare and the coexistence of malignant and benign tumors is rarer still. Additionally, the association of fibrolamellar hepatocellular carcinoma and auricular myxoma is scarcely reported in the literature. In our case, our patient was treated in accordance with the established surgical procedures, received oral doses of IMMUNEPOTENT CRP, and, as of this writing, is completely asymptomatic.
